# Muscle creatine levels and sprint performance in young adult vegans and vegetarians after 7 days of creatine monohydrate supplementation

**DOI:** 10.14814/phy2.70539

**Published:** 2025-09-12

**Authors:** Thomas Christian Bonne, Valentina Arthemalle, Colin Stephen Doherty, Andreas Breenfeldt Andersen, Jacob Bejder, Thomas Ehlers, Henrik Holm‐Sørensen, Nikolai Baastrup Nordsborg

**Affiliations:** ^1^ Department for Nutrition, Exercise and Sports University of Copenhagen Copenhagen Denmark; ^2^ School of Medical and Health Sciences Edith Cowan University Perth Western Australia Australia; ^3^ Department of Public Health, Research Unit for Exercise Biology Aarhus University Aarhus Denmark; ^4^ Department of Anaesthesiology Herlev & Gentofte Hospital Herlev Denmark

**Keywords:** muscle creatine, sprint performance, vegans, vegetarians

## Abstract

Athletes use creatine monohydrate (CM) to enhance high‐intensity exercise performance by increasing creatine phosphate availability. While CM supplementation is known to raise muscle creatine levels in vegans and vegetarians, its impact on exercise performance remains uncertain. We examined the effects of CM supplementation on muscle creatine content and exercise performance in vegans and vegetarians. In a randomized, double‐blind placebo‐controlled design, 15 healthy vegans and vegetarians consumed CM (0.3 g/kg/day, *n* = 7) or placebo (PLA, 0.3 g maltodextrin/kg/day, *n* = 8) four times a day for 7 days. Before and after supplementation, repeated sprint capacity was determined. Body mass and fat‐free mass (FFM) were assessed by dual‐energy x‐ray absorptiometry. The CM group increased body mass (1.56 ± 0.57 kg, *p* < 0.01) and FFM (1.15 ± 0.94 kg, *p* < 0.05), while the PLA group showed no changes. In the CM group, muscle creatine (Cr) and total muscle creatine (TCr) increased by 18.8 ± 13.1 mmol/kg (*p* < 0.05) and 30.8 ± 21.2 mmol/kg (*p* < 0.01), respectively. The PLA group showed no changes in Cr and TCr (−4.6 ± 13.1 mmol/kg and 2.9 ± 11.6 mmol/kg, respectively). Phosphocreatine levels remained consistent within and between groups. There were no observed changes in peak and mean power output during repeated sprints. A seven‐day CM supplementation in healthy vegans and vegetarians increased Cr and TCr whereas Phosphocreatine, peak and mean power output during repeated sprints was unchanged.

## 1 INTRODUCTION

Diets with a high proportion of plant matter, rich in fiber and phytochemicals, are positively correlated with overall health (Le & Sabaté, 2014; Melina et al., 2016; Venderley & Campbell, 2006) and represent a sustainable food choice (Willett et al., 2019). As a result, there has been an increase in the number of vegan and vegetarian (VEG) consumers (Craddock et al., 2016; Melina et al., 2016; Rogerson, 2017), including those in athletic populations (Kamiński et al., 2020; Leitzmann, 2014). However, due to the altered macronutrient composition of plant‐based diets, particularly the lower protein content, special attention may be required for competitive athletes (Burke et al., 2003). Despite this, studies have shown that VEG have similar performance outcomes to omnivores (Khanna et al., 2006; Rogerson, 2017). It is worth noting that plant‐based protein often lacks essential amino acids such as leucine, tryptophan, and methionine (Shomrat et al., 2000). Of particular importance is methionine, which is a precursor for creatine synthesis found in meats, fish, and poultry. Consequently, VEG typically have lower dietary and muscle creatine levels (Greenhaff et al., 1993).

Creatine supplementation efficiently increases muscle creatine phosphate (PCr) stores (Forbes et al., 2023), and more so in VEG (Kaviani et al., 2020). Moreover, creatine supplementation improves repeated short‐term high‐intensity exercise performance (Cooper et al., 2012; Greenhaff et al., 1993; Kreider et al., 2017). However, evidence of creatine supplementation in VEG for improving a single bout short duration high‐intensity performance is limited and equivocal. In a cross‐sectional study, omnivores outperform VEG on a 30 s all‐out cycling test (Shomrat et al., 2000), suggesting the potential benefit of PCr supplementation for VEG. However, neither VEG nor omnivores experienced improved single 30 s all‐out sprint following creatine supplementation (Odland et al., 1997; Snow et al., 1998; Watt et al., 2004) although other studies have shown a positive effect (Birch et al., 1994; Earnest et al., 1995). When evaluating the potential effect of PCr supplementation on intense exercise performance, exercise duration is likely of high importance. Sprinting for ≤10 s can deplete PCr levels by more than 50% of resting levels (Gaitanos et al., 1993). During 20 to 30 s sprints, PCr depletion can reach 73% and 83%, respectively, but the relative importance of glycolytic and oxidative phosphorylation energy contribution increases substantially (Cottrell et al., 2002; Gaitanos et al., 1993; Green et al., 1996; Harris et al., 1992; Hultman et al., 1996). Thus, evaluating the effect of creatine supplementation in VEG populations performing 10–15 s sprinting may improve the sensitivity for detecting potentially relevant changes.

Importantly, recent longitudinal evidence suggests that, when total protein intake is matched and optimized (e.g., 1.6 g·kg^−1^·day^−1^), a high‐protein exclusively plant‐based diet supports muscle mass and strength gains comparable to an omnivorous diet. Hevia‐Larraín et al. (2021) found no significant differences in leg lean mass, muscle cross‐sectional area, or strength outcomes between habitual vegans and omnivores following a 12‐week supervised resistance training program, provided both groups consumed adequate protein via whole foods and supplemental protein. These results challenge the notion that plant‐based protein sources are inferior for supporting resistance training adaptations.

Hence, we designed a study to assess the effects of creatine supplementation on performance measures in a strict vegan or vegetarian population. The hypothesis was that creatine supplementation increases muscle total creatine (TCr) content as well as 15 s all‐out sprint performance.

## 2 METHODS

### 2.1 Study design

This study was approved by the Copenhagen Ethics Committee (H‐19043974) and performed in accordance with the declaration of Helsinki (“World Medical Association Declaration of Helsinki,” 2013). The investigation was conducted at the Department of Nutrition, Exercise and Sports, University of Copenhagen. Participants were informed about the experiment and potential risks, verbally and in writing, with the option to withdraw at any time. Written informed consent was obtained for all participants before the start of the study. All participants completed two familiarization trials of the entire exercise protocol on two separate days. Next, baseline measurements were completed and followed by a seven‐day supplementation period. Participants were randomly assigned to receive either creatine monohydrate (CM) or maltodextrin as placebo (PLA). Post‐supplementation testing, which was identical to the baseline testing, was conducted 24–48 h (31 ± 7) after the supplementation period. Randomization to CM and PLA was performed by an investigator not involved in study design or data collection. The allocation sequence was determined by a computerized random number generator (www.randomizer.at). Participants did not learn of their supplement group until 1–2 weeks post‐supplementation. Investigators were informed ~2 days after the last performance test. Participants were instructed to maintain regular physical activity and dietary intake over the 7‐day supplementation period. Physical activity was tracked through the long‐form International Physical Activity Questionnaire (IPAQ) (Fogelholm et al., 2006) to confirm self‐reported exercise duration and frequency.

### 2.2 Participants

Fifteen participants (6 males and 9 females) were enrolled (Table 1). All participants reported that they engaged in a minimum of 30 to 60 min of physical activity three times a week and followed a vegan/vegetarian diet. Participants were verbally screened for conditions and/or medication that may affect creatine metabolism, absorption and/or impair their ability to perform any of the exercise tests. To ensure accurate self‐classification, all participants were provided with a comprehensive explanation of the dietary definition of a vegan and vegetarian diet (Weinsier, 2000). Additionally, an unweighted seven‐day food diary was collected to confirm self‐reported dietary classification. A vegan diet was defined as *exclusively* plant‐based foods with the exclusion of all animal foods as well as food products that contain them. A vegetarian diet was defined as *predominantly* plant‐based foods with the inclusion of dairy products, eggs and honey but abstaining from meat, poultry, seafood, and all animal flesh.

**TABLE 1:** Anthropometric measurements by DXA‐scan before and after the intervention.

	PLA (*n* = 8) (♀4, ♂4) (vegans = 6, vegetarians = 2)		CM (*n* = 7) (♀5, ♂2) (vegans = 5, vegetarians = 2)	
	Pre	Post	Pre	Post
Age	24.5 ± 3.2		22.7 ± 2.6	
Height (cm)	174.4 ± 6.2		170.4 ± 8.0	
Weight (kg)	69.8 ± 7.4	69.8 ± 7.8	67.9 ± 11.0	69.5 ± 11.0**
Fat mass (kg)	14.1 ± 4.3	13.9 ± 4.2	15.5 ± 3.6	15.9 ± 3.4
Fat‐free mass (kg)	56.9 ± 10.2	57.2 ± 10.2	52.4 ± 9.5	53.6 ± 10.1*

*Note*: Statistically significant differences: *p < 0.05, **p < 0.01.

### 2.3 Supplementation protocol

Premeasured amounts of CM (MyProtein, UK) or PLA (maltodextrin, MyProtein, UK) in powder form were supplied to each participant (0.3 g/kg/day) depending on group allocation by an investigator not involved in the performance tests. This regime is typical of a loading phase used in omnivores and is assumed to facilitate maximal uptake in vegans/vegetarians (Harris et al., 1992; Hultman et al., 1996). The CM and placebo powder were identical in appearance and texture. Following the baseline measurements, each participant received a package containing 28 individual sachets. All participants were instructed to consume four packages per day (breakfast, lunch, dinner and pre‐bed) with approximately 500 mL of water and a carbohydrate source to promote muscle uptake (Green et al., 1996). After 7 days of supplementation, they returned their empty packages as a surrogate measure of compliance. All subjects reported consuming 28/28 packages with two exceptions, one in the CM and PLA group (27/28 and 27/28).

### 2.4 Body composition

Body mass and height were measured on a scale accurate to the nearest 0.1 kg and 0.1 cm, respectively. Subjects were weighed in a t‐shirt/crop‐top, shorts/leggings and socks at the same approximate time of day (±2 h) pre‐ and post‐supplementation. Body mass and height were measured twice to ensure the accuracy of the measurement, and if the two measures differed, the highest value was recorded. Participants' body composition was analyzed using dual‐energy x‐ray (DXA) absorptiometry (Lunar iDXA, GE Healthcare, Madison, WI, USA) to determine fat‐free mass and body fat mass. Baseline and follow‐up scans occurred at the same time of day (±2 h) with an empty bladder. The software determines fat‐free mass from lean tissue mass and bone mineral content assuming a constant hydration level (approximately 73%). Participants were instructed to consume approximately 500 mL of water 2 h prior to testing to help standardize fluid intake and empty both the bladder and bowel if needed before the DXA scan.

### 2.5 Muscle biopsies

Percutaneous needle biopsies were obtained from the m. vastus lateralis under local anesthesia, with 1% lidocaine and suction applied via a 60‐cc syringe using the Bergström technique (Bergström, 1975). Target biopsy time after participants post‐supplementation exercise performance test was 24 h. Pre‐ and post‐biopsies were immediately immersed in −80°C liquid nitrogen.

A portion of muscle (10 to 15 mg wet weight; ww) was freeze‐dried for approximately 48 h, crushed, and weighed (2 mg). Every sample was analyzed in duplicates. Powdered samples were extracted using 3N perchloric acid (PCA), neutralized with 2N potassium hydrogen carbonate (KHCO_3_) and assayed for PCr and Cr concentrations using enzymatic analysis with fluorometric detection (Burke et al., 2003). TCr content was determined as the sum of Cr and CrP.

### 2.6 Repeated sprint ability and capillary sampling

Participants began with a standard bike fitting procedure for seat height and shoe size, which was followed by a 4‐min warm‐up at ~80 rpm against a resistance of 1 kg for females and 1.5 kg for males on a cycle ergometer (Monark 894 E Peak Bike, Weight Ergometer, Vansbro, Sweden). Immediately after the warm‐up, the first capillary sample was collected during a 3‐min recovery period against zero resistance, using 95 μL tubes (Clinitubes, Radiometer, Denmark) and analyzed for lactate, pH, and HCO_3_^−^ on a dedicated blood gas analyzer (ABL800 Flex, Radiometer, Denmark). Next, the participants performed four 15 s all‐out sprints interspersed by 3‐min recovery periods. The sprints were performed at the participant's maximal cadence against a flywheel resistance relative to their body mass at baseline (0.075 kg/body). Before each sprint, prior to applying resistance to the flywheel, participants were allowed 3 s to increase speed from a standstill, providing a flying start. Equivalent verbal encouragement was provided to all participants throughout each sprint. Capillary samples from one of the five fingers on the left hand were collected immediately after each sprint. Post‐supplementation testing followed identical procedures as pre‐supplementation. Peak power output was defined as the maximum power output recorded by the Monark Anaerobic Test Software (Vansbro, Sweden) during any of the 15 s sprints. Overall peak power output was the average of the peak power values for each 15 s sprint. Mean power output was the mean power maintained during each 15 s sprint. Overall mean power output was the average of the mean power output maintained for each 15 s sprint.

### 2.7 Statistical analysis

Based on power calculations, a sample size of 14 participants was needed to achieve a statistical power of 0.8 with an α of 0.05. The sample size calculated was based on a study from Solis and Gualano (Solis et al., 2017) who presented changes in muscle PCr following creatine loading (0.3 g/kg/bw for 7 days) in vegetarian adults. This study was selected for power analysis as it utilized the exact loading protocol and duration applied in the present study. The coefficient of variation (CV%) for Cr, PCr and performance outcomes was calculated as the standard deviation of the absolute differences in the PLA group Pre and Post the treatment period divided by the grand mean and dividing the quotient by √2. A mixed model analysis with “Time” (Pre, Post) “treatment” (CM, PLA) and time×treatment as fixed factors, with subject ID to identify repeated measures and to define a random factor, was performed in IBM SPSS v. 28.0.0 statistical software package. A post hoc analysis was performed if a significant main effect was observed using the Holm‐Sidak adjustment for multiple comparisons. Unless otherwise stated, results are mean ± SD. Significance was set at *p* < 0.05.

## 3 RESULTS

### 3.1 Body composition

Age, body mass, and height were similar between groups at baseline (Table 1). A time × treatment effect (*p* < 0.05) for body mass and fat‐free mass was seen, but differences between groups were not detectable. The supplementation protocol increased body mass (1.56 ± 0.57 kg, *p* < 0.01) and fat‐free mass (1.15 ± 0.94 kg, *p* < 0.05) significantly in the CM group. No changes were observed in the PLA group.

### 3.2 Muscle creatine content

**FIGURE 1:**
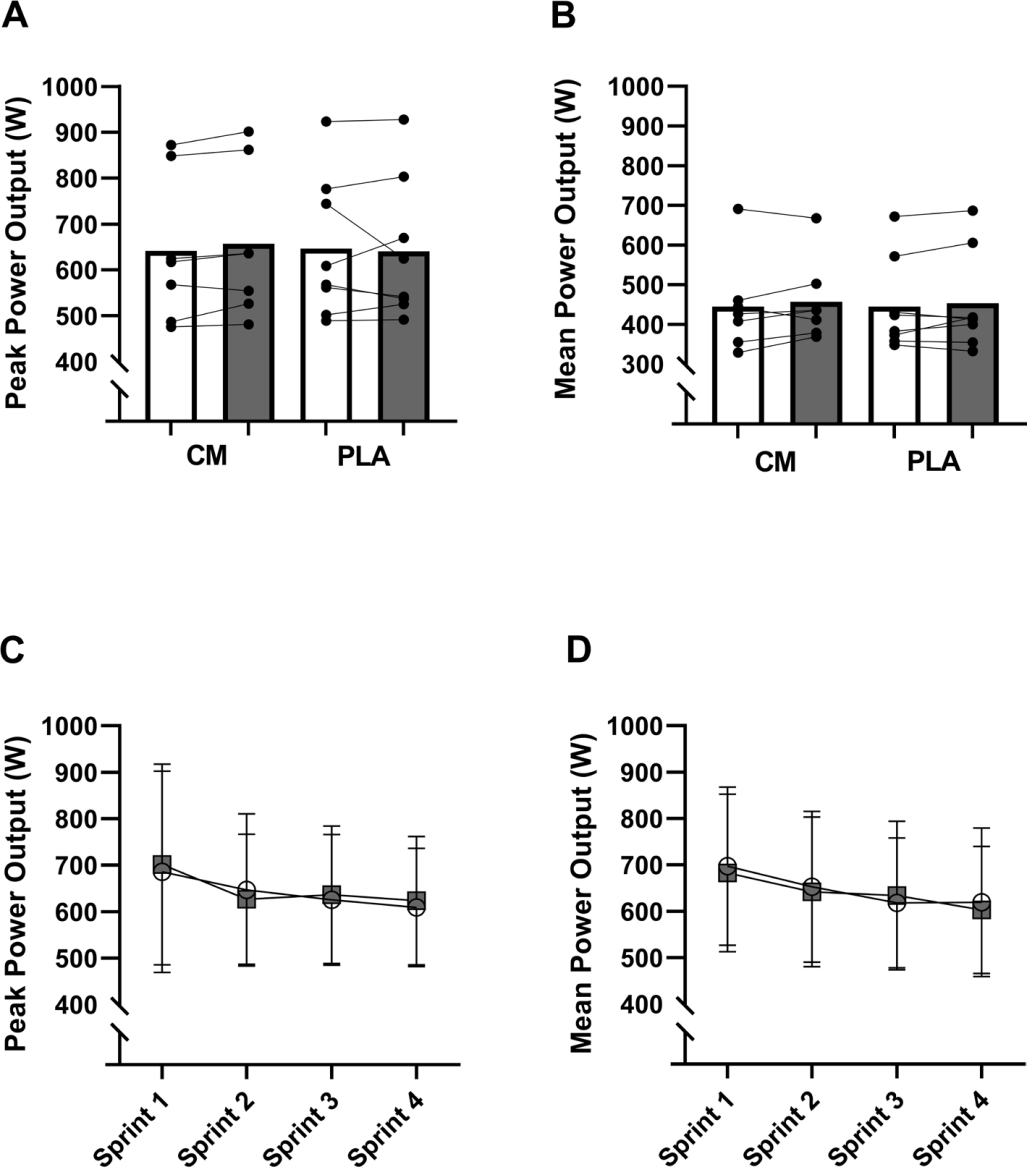
Muscle tissue was analyzed for (a) creatine phosphate (CrP), (b) creatine and (c) TOTAL creatine for the creatine monohydrate supplementation group (CM) and placebo (PLA), respectively. White bars represent pre levels while gray bars represent post‐intervention values. Values are means and error bars indicate one standard deviation. Symbols in brackets indicate the statistical differences from pre to post.

The CV% of all duplicate measurements for Cr and PCr was 1.1%. A significant time × treatment interaction was detected for muscular Cr levels (*p* < 0.05). The CM group increased Cr level by 18.8 ± 13.1 mmol/kg (*p* < 0.05) whereas no change (−4.6 ± 13.1 mmol/kg) was detected in the PLA group (Figure 1). While the baseline levels of muscular Cr were similar between groups at baseline, the CM group had 25.8 ± 19.1 mmol/kg higher (*p* < 0.01) level post‐supplementation. There were no main effects on intramuscular PCr levels (Figure 1).

TCr levels displayed a significant time × treatment effect (*p* < 0.05) with an increase of 30.8 ± 21.2 mmol/kg (*p* < 0.01) in the CM group, whereas it was unaltered in (2.9 ± 11.6 mmol/kg) PLA. As with Cr levels, the TCr levels were similar between groups at baseline, but the CM group had a 37.1 ± 25.8 mmol/kg higher (*p* < 0.001) TCr level post‐supplementation (Figure 1).

### 3.3 Repeated sprint ability and capillary blood

**FIGURE 2:**
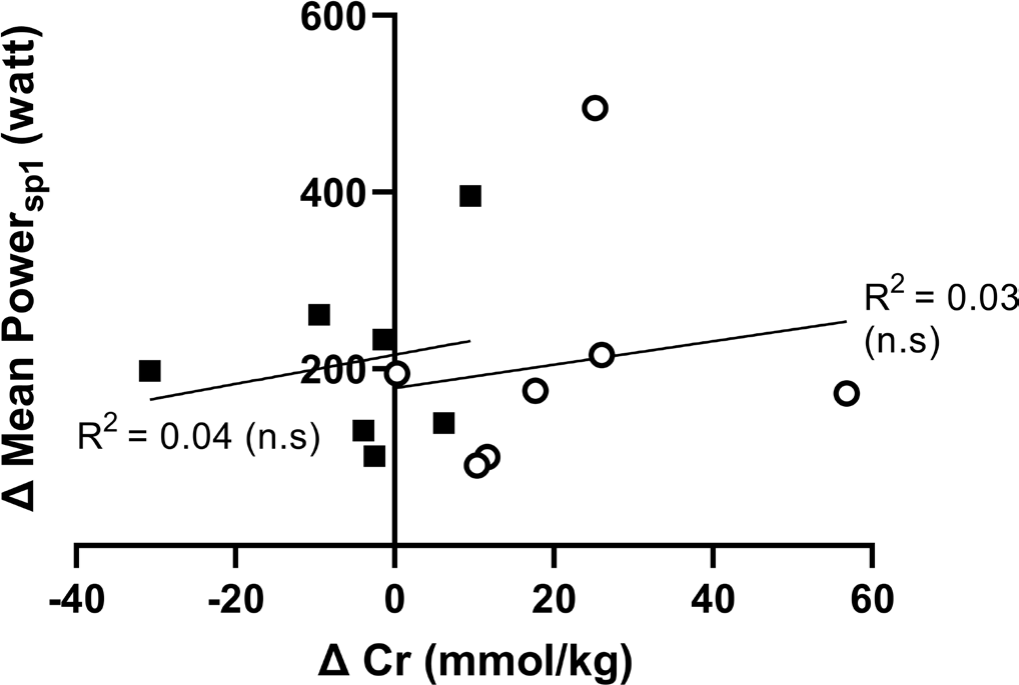
Power output from four repeated 15‐second sprints. (a) The highest recorded peak power output measured from any of the four sprints and (b) The mean power output from all four sprints for the creatine monohydrate group (CM) and placebo group (PLA). Bars represent the group mean while lines represent individual values. White bars are pre‐intervention values with gray bars representing post‐values. (c) Peak power output for each sprint for CM and (d) PLA group. Open circles represent pre‐values with filled squares representing post‐values.

The CV% for mean power output during all four 30s sprints in PLA was 2.2%. There was no main effect (time × treatment × sprint) on either peak power or mean power output for the individual sprint intervals or combined mean of all intervals (Figure 2). The CM group significantly decreased power from the first to the fourth sprint both pre and post (77 ± 110 watt and 78 ± 110 W for pre and post in the CM group respectively, both *p* < 0.05) while the PLA group only reduced peak power from the first to the fourth sprint post‐supplementation (80 ± 82 W, *p* < 0.01). The time × treatment × sprint interaction was not significant for capillary lactate, pH or HCO_3_^−^ (Table 2).

**TABLE 2:** Obtained values from capillary blood samples for lactate, pH, and HCO3^−^ for the creatine monohydrate supplementation group (CM) and placebo group (PLA).

Group	Timepoint	Sprint	Lactate	pH	HCO_3_^−^
CM	Pre	S0	3.3 ± 0.4	7.4 ± 0.0	23.1 ± 1.1
		S1	6.7 ± 1.7	7.3 ± 0.0	20.5 ± 1.7
		S2	10.3 ± 1.3	7.3 ± 0.0	17.3 ± 1.7
		S3	14.8 ± 2.8	7.2 ± 0.0	13.9 ± 1.9
		S4	16.7 ± 2.5	7.2 ± 0.0	12.8 ± 2.0
	Post	S0	3.3 ± 1.2	7.4 ± 0.0	24.1 ± 1.8
		S1	6.3 ± 1.1	7.3 ± 0.0	22.0 ± 1.5*
		S2	10.9 ± 2.3	7.3 ± 0.0	17.7 ± 1.7
		S3	13.9 ± 2.4	7.2 ± 0.0	14.7 ± 2.0
		S4	16.6 ± 3.2	7.2 ± 0.1	12.2 ± 2.4
PLA	Pre	S0	4.4 ± 2.5	7.4 ± 0.1	21.6 ± 3.3
		S1	8.0 ± 1.8	7.3 ± 0.0	19.5 ± 2.0
		S2	13.4 ± 2.8	7.2 ± 0.0	15.3 ± 2.0
		S3	16.1 ± 3.5	7.2 ± 0.0	13.1 ± 1.9
		S4	17.5 ± 3.9	7.2 ± 0.0	11.6 ± 2.3
	Post	S0	3.9 ± 3.0	7.4 ± 0.0*	22.3 ± 3.3
		S1	6.6 ± 2.7	7.3 ± 0.0	20.5 ± 3.0
		S2	11.1 1.9*	7.3 ± 0.0*	15.9 ± 2.2
		S3	14.4 2.6*	7.2 ± 0.0	13.0 ± 2.1
		S4	15.8 3.0*	7.2 ± 0.0	11.6 ± 2.4

*Note*: S0, 1, 2, 3, and 4 denote a measurement after warm‐up, sprint 1, sprint 2, sprint 3, and sprint 4, respectively. Statistically significant differences: *p < 0.05.

## 4 DISCUSSION

The aim of this study was to investigate the hypothesis that creatine supplementation in a vegan and vegetarian population would lead to an increase in TCr in skeletal muscle and an associated improvement in short‐term exercise performance. The primary finding was that 7 days of creatine monohydrate (CM) supplementation successfully increased TCr and creatine levels, whereas intense exercise performance remained unaltered.

### 4.1 Muscle creatine content body composition in VEG after creatine supplementation

Creatine supplementation increases muscular TCr and PCr (Cooper et al., 2012; Kreider et al., 2017; Mielgo‐Ayuso et al., 2019). In the current study, we were unable to confirm these findings regarding PCr changes in VEG, whereas TCr was increased after 7 days of CM loading. The lack of a significant increase in PCr may be related to PCr‐hydrolysis as an adverse effect of the biopsy procedure (Janssen et al., 2016). A prevalent observation in human muscle studies examining creatine uptake through biopsy analysis is that TCr levels tend to rise more than phosphocreatine PCr (Hultman et al., 1996; McKenna et al., 1999) whereas non‐invasive studies have not found the same discrepancy (Brault et al., 2007; Janssen et al., 2016). It should, however, be noted that a larger sample size than the current may have yielded a different result.

Comparative analysis of muscle creatine levels between omnivorous and vegetarian individuals has unveiled notable distinctions. Individuals adhering to an omnivorous diet, characterized by regular consumption of animal‐derived foods rich in creatine, exhibit higher levels of muscle creatine in contrast to their vegetarian counterparts (Lukaszuk et al., 2002). While endogenous synthesis contributes to overall creatine levels, the reduced availability of exogenous creatine from dietary sources among VEG remains a key determinant in the observed differences. Vegetarians have baseline muscle creatine levels of ~100 mmol/kg of dry muscle on average compared to ~120 mmol/kg in omnivores (Kreider et al., 2017) The lower baseline creatine content may explain why vegetarians experience a greater absolute muscle creatine increase after supplementation with creatine monohydrate (Benton & Donohoe, 2011; Brosnan & Brosnan, 2016). Multiple research groups have reported higher increases in gastrocnemius PCr (Solis et al., 2017), vastus lateralis TCr (Blancquaert et al., 2018; Watt et al., 2004), erythrocyte creatine content (Shomrat et al., 2000) and plasma creatine concentrations (Blancquaert et al., 2018; Delanghe et al., 1989) following supplementation in vegetarians compared to an omnivore or control group. Surprisingly, the CM average baseline TCr level of 114 ± 17 mmol/kg in the present study resembles that previously reported for omnivores, and with supplementation, the VEG TCr content approached the level of an apparent muscle threshold (~160 mmol/kg) as determined in omnivores (Kreider et al., 2017). This raises the question of whether adherence to a strict vegan or vegetarian diet was upheld in the current study. However, there were no indications of participants not adhering to their reported diet, which was monitored through self‐reported food diaries. Another explanation could be that the participants in the present study were skilled in selecting food sources providing protein of higher quality from a plant‐based diet (Fuhrman & Ferreri, 2010; Wirnitzer et al., 2018), but that remains speculative. In addition, a previous intervention consisting of 21 days of a lacto‐ovo‐vegetarian diet showed large inter‐individual differences in muscle creatine content (Lukaszuk et al., 2002). Nevertheless, taken together, the muscle tissue analyses clearly demonstrate the expected effect of the supplementation protocol, namely an increased muscle creatine content.

Concomitantly, body mass and fat‐free mass increased, supporting the muscle biopsy analysis. It should be noted, though, that water retention is a well‐documented side effect of creatine supplementation and can introduce bias when interpreting changes in body composition or body mass. This fluid retention may confound results in studies using DXA or bioelectrical impedance analysis, which cannot always distinguish between water and true muscle hypertrophy (Francaux & Poortmans, 1999). Therefore, the difference we see in fat‐free mass can be attributed to water retention.

### 4.2 Short term sprint performance after creatine supplementation in VEG

In the present study, there were no significant differences between groups when assessing measures of exercise performance: peak power, mean power, and concentration of capillary blood metabolites, despite the elevated muscle TCr level. The lack of effect of creatine loading on exercise performance in omnivores and vegetarians has previously been observed (Kaviani et al., 2020; Watt et al., 2004). Specifically, Watt et al. (2004) utilized two 30 s Wingate sprints separated by a 4 min rest period following a 5‐day CM loading period (0.4 g/kg/day) in a vegetarian and an omnivorous group. Despite a greater vastus lateralis TCr increase (~41%) in the vegetarian population, participants did not improve initial 30 s mean power output. It should be noted that mean power output during the second Wingate test did increase. It should be noted that the study was powered to detect a difference in muscle creatine. If the power analyses were based on detecting peak and mean power output differences from pre to post, a post‐study power analysis revealed the required number of subjects to be *n* = 23. Hence, the results from repeated sprints should be interpreted accordingly. Shomrat et al. (2000) implemented the most comparable exercise protocol to the one utilized in the present study (3 × 20 s maximal cycling, interspersed by 4 min recovery). Seven male vegetarians and nine omnivores received a loading dose (3 × 7 g creatine/day for 6 days), where both mean power and peak power were analyzed. Both the vegan/vegetarian and omnivore groups experienced improved performance after supplementation and, importantly, demonstrated improved sprint ability in the first 20 s exercise bout. In contrast, we did not detect any differences in mean power after supplementation. Both Shromat et al, and the present study implemented long recovery periods (3 and 4 min) in between sprints. Shomrat and colleagues utilized 5.2% of body mass on the cycling ergometer flywheel while the present study and Watt et al. utilized 7.5%.

Burke et al. (2003) designed the best example of a rigorously controlled randomized trial for investigating the relationship between muscle creatine and exercise performance in vegetarians to date. Although it is the least comparable to the present study, important findings were reported. Four groups were assessed over 8 weeks: 1) vegetarian‐placebo (*n* = 8); 2) vegetarian‐creatine (*n* = 10); 3) omnivore‐placebo (*n* = 13); and 4) omnivore‐creatine (n = 13). Concurrent supplementation and a high‐volume resistance training program were implemented for each group. The vegetarian‐creatine group had the greatest increase in vastus lateralis TCr (~30%) and PCr (~66%), which translated to a fat‐free mass increase (2.4 kg vs. 1.9 kg in the omnivore‐creatine group) by the end of the 8‐week intervention. This is one of the only studies where the higher muscle TCr and PCr in vegetarians translated to enhanced performance, and one of the only studies that utilized an exercise intervention and creatine supplementation simultaneously. The vegetarian group increased work performed (~30%) during a 50‐repetition maximal knee extension exercise compared to the omnivore group, which only marginally increased (~9%). The vegetarian group that supplemented with creatine had a greater increase in TCr, PCr, lean tissue, and total work performed compared to omnivores who supplemented with creatine. This is not directly comparable to the present study due to the high‐volume resistance program, duration, supplement regime, sample size, and populations investigated. However, it does provide strong evidence that resistance training may serve as a catalyst for muscular adaptations and subsequently improve performance outcomes when supplementing with creatine in vegetarians. Therefore, creatine may provide a small benefit in isolation, but when coupled with resistance exercise, the benefits are potentiated.

Nevertheless, peak power output improvement has been reported in omnivores for one study (Shomrat et al., 2000).

**FIGURE 3:**
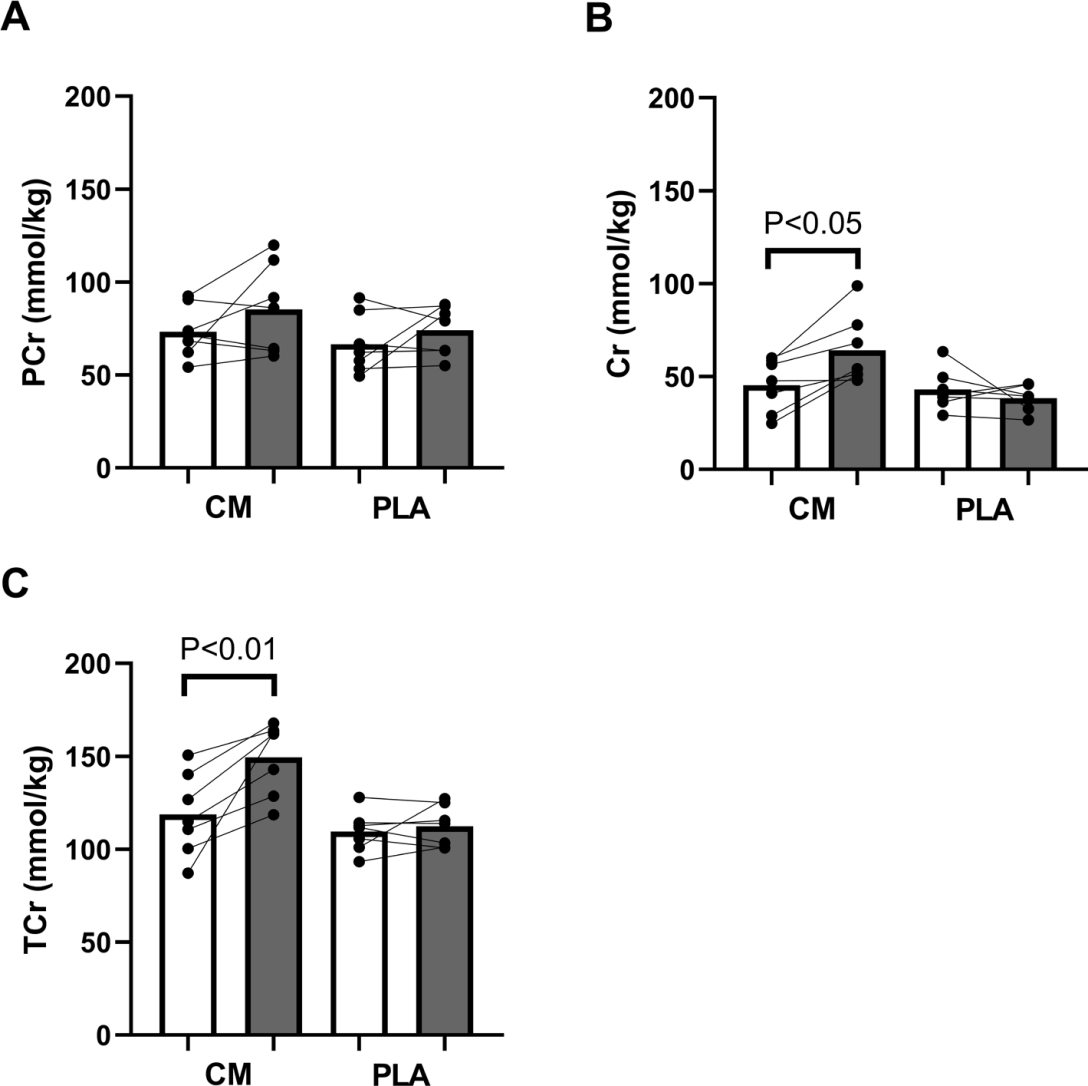
Correlation between changes from pre‐supplementation to post‐supplementation for muscular creatine (∆ Cr) and changes in mean power output (∆ Mean Power Output_Sp1_) for the first sprint. Open circles represents the CM group while black squares represents the PLA group.

It must be considered if the lack of effect on sprint performance in VEG is due to variability in the response to creatine loading and/or the familiarity to repeated sprinting. It is well‐described that muscular creatine increases vary between individuals (Lukaszuk et al., 2002). In Figure 3, it is clear that a few individuals in the supplementation group experienced elevated creatine levels and improved sprinting ability, while others had low increases of muscular creatine as well as no detectable effect on sprint performance. However, the relatively low number of participants does not allow for an adequate analysis of the potential individual response to creatine loading with regard to muscle content and sprint ability.

Unfortunately, the research is limited in vegan and vegetarian populations, particularly for exercise performance outcomes, with a large degree of heterogeneity between trials (R. C. Harris et al., 1992; Hultman et al., 1996).

## 5 STRENGTHS AND LIMITATIONS

The strengths of this study are the randomized, double‐blind, placebo‐controlled design, which should account for potential confounding while also minimizing bias. Self‐reported exercise and diet were confirmed via interviewer‐led questionnaires. Data was collected on diet duration, which is typically not seen in these investigations. Although it may be of importance, as a participant who has practiced a vegan/vegetarian diet for 6 months may respond differently than an individual who has been practicing the diet for 10 years. Hence, there is a need for increased sample sizes and thereby adequately powered studies to facilitate stratification and analysis by diet group (vegan, vegetarian) and duration (i.e., 6 months, 5–10 years), to determine whether diet group or duration serves as mediating factors for muscle creatine and/or exercise performance.

However, self‐reporting of diet also introduces significant limitations when characterizing vegans and vegetarians, primarily due to misclassification and reporting bias. Individuals may inaccurately label themselves as vegan while occasionally consuming animal products, either intentionally or due to misunderstanding dietary definitions. Social desirability bias can also lead participants to underreport animal product consumption or overreport adherence to plant‐based foods.

Power output has been shown to be significantly higher during the day compared to the morning, which is attributed to the fluctuations in body temperature, coordination, and neural drive (Teo et al., 2011). Pre‐ and post‐supplementation measures were nearly identical with regard to the time of day and day of testing, thereby reducing the biological variability of circadian rhythms (Teo et al., 2011).

The limitations of this study include the lack of an omnivore comparator group to determine if the changes in TCr can be expected to be bigger in a VEG group compared to omnivores, although it seems unlikely given that we did see an effect of loading on body weight and TCr in VEG. Additionally, the performance changes between VEG and omnivores after loading need to be thoroughly examined. A lack hereof is important to establish since loading, with a concomitantly increased body weight, might impact performance negatively.

The study was initially powered to detect a difference in vegetarian muscle TCr from pre to post‐supplementation. If the power analyses were based on detecting peak and mean power output differences from pre to post, a post‐study power analysis revealed the required amount of subjects to be *n* = 23.

## 6 CONCLUSION

The current study clearly demonstrates that a seven‐day creatine monohydrate loading phase in recreationally active vegan and vegetarian adults increases muscle TCr but not PCr content. Despite the increased potential for anaerobic alactid energy provision, peak and mean power output during repeated sprint cycling did not improve when the sprint durations and recovery time were 15‐s and 3‐min, respectively.

## FUNDING INFORMATION

No funding information provided.

## CONFLICT OF INTEREST STATEMENT

The authors declare no conflict of interest. The results of the study are presented clearly, honestly, and without fabrication, falsification, or inappropriate data manipulation.

## ETHICS STATEMENT

This study was approved by the Copenhagen Ethics Committee (H‐19043974) and performed in accordance with the declaration of Helsinki.

## Data Availability

Data is available upon request with the appropriate data sharing agreement.
